# The Time Burden of Specialty Clinic Visits in Persons With Neurologic Disease: A Case for Universal Telemedicine Coverage

**DOI:** 10.3389/fneur.2021.559024

**Published:** 2021-05-04

**Authors:** Daniel L. Solomon, Benjamin Dirlikov, Kazuko L. Shem, Christopher S. Elliott

**Affiliations:** ^1^Rehabilitation Research Center, Santa Clara Valley Medical Center, San Jose, CA, United States; ^2^Department of Physical Medicine and Rehabilitation, Santa Clara Valley Medical Center, San Jose, CA, United States; ^3^Division of Urology, Santa Clara Valley Medical Center, San Jose, CA, United States; ^4^Department of Urology, Stanford University School of Medicine, Stanford, CA, United States

**Keywords:** telemedicine, burden, neurological disorders, disability, caregiver, commute time

## Abstract

**Objective:** Those with chronic neurologic disorders are often burdened not only by the condition itself but also an increased need for subspecialty medical care. This may require long distance travel, while even small distances can be a hardship secondary to impaired mobility and transportation. We sought to examine the burden of time associated with clinical visits for those with chronic neurologic disorders and their family/caregivers. These topics are discussed as an argument to support universal coverage for telemedicine in this population.

**Design:** Cohort Study.

**Setting:** Specialty clinic at community hospital.

**Participants:** 208 unique patients with chronic neurologic disability at physical medicine and rehabilitation or neurourology clinic over a 3-month period.

**Main Outcome Measures:** Patient survey on commute distance, time, difficulties, and need for caregiver assistance to attend visits.

**Results:** Approximately 40% of patients were covered by Medicare. Many patients (42%) perceived it difficult to attend their clinic visit with transportation difficulties, commute time, and changes to their daily schedule being the most commonly cited reasons. Most patients (75%) lived within 25 miles of our clinics and experienced an average commute time of 79.4 min, though 10% required 3 h or more. Additional family/caregiver assistance was required for 76% of patients, which resulted in an inclusive average commute time of 138.2 min per patient.

**Conclusion:** Chronically neurologically-disabled patients and their caregivers may be burdened by the commute to outpatient appointments. To minimize this burden, increased emphasis on telemedicine coverage for those with chronic neurologic disability should be considered by all payors.

## Introduction

With the advent of subspecialty care for persons with chronic neurologic disorders, there have been substantial efforts to improve both health outcomes and patient satisfaction (1–3). However, persons afflicted with chronic neurologic disorders often have dysfunction in more than one organ system requiring subspecialty care from multiple providers on an ongoing basis. In many instances, for those with chronic neurologic disability, care is not available in a multidisciplinary fashion and leads to multiple clinic visits on varying days (4). In addition, specialty physicians are not always available in a patient's local community, which can result in long distance travel to obtain appropriate care (5, 6). For many persons with chronic neurologic disability, the time spent attending repeated medical visits can disrupt their personal and professional lives, and results in a significant ongoing burden to them and their caregivers (7, 8).

In recent years, to address barriers to care, telemedicine has been introduced to provide convenient patient care (4, 8–13). As seen during the COVID-19 outbreak, telemedicine has expanded to include live synchronous audiovisual conference, store-and-forward videoconferencing, remote patient monitoring, and general education information (14). Compared to traditional in-person medical visits, telemedicine visits can be performed remotely, often from the convenience of a patient's home or place of employment (15–17). Overall, patient and clinician satisfaction with live telemedicine visits are reported to be high with comparable health related outcomes to in-person visits (8, 10, 18–20).

However, outside of temporary changes brought about by the COVID-19 pandemic, telemedicine is still not a universally patient-covered benefit and reimbursement for telemedicine, even if covered, is not always straightforward. For example, Medicare beneficiaries with neurologic disorders (including, individuals over 65 years old with Medicare coverage, individuals <65 years old receiving Social Security disability as a result of a debilitating neurologic condition, or individuals with amyotrophic lateral sclerosis), receive restrictive telemedicine coverage (21). Specifically, for telemedicine to be covered by Medicare, the beneficiary must physically be in a health care clinic or inpatient hospital setting, and also be in a rural location where access to a specialist may be unavailable (22). Currently, once coronoavirus-19 pandemic provisions expire, Medicare will not cover telemedicine visits with their specialists if the individuals are at home. As a result, those with chronic neurologic disease and their caregivers may continue to require substantial amounts of time to coordinate and travel to in-person visits with their physicians.

To date, the overall “time burden” that is shouldered by those with chronic neurologic disability to physically attend clinic visits in person has been incompletely evaluated. A greater understanding of the time burden and the patient experience may facilitate the promotion of telemedicine coverage. This study examined the time burden and difficulties of patients and their caregivers attending clinic visits in a specialty center.

## Methods

We conducted a survey-based quality improvement project at a community specialty outpatient clinic in Northern California from January 1, 2019 to March 1, 2019. The study was deemed a quality improvement project and was exempt from Institutional Review Board review. During the study period, all patients with chronic neurogenic disorders receiving outpatient clinical care from either the Department of Physical Medicine and Rehabilitation or Division of Urology were administered a twenty-question paper survey regarding distance traveled, transportation difficulties, and the time required for the patients and their caregiver to prepare and commute to their appointments (Supplementary Material 1). In cases where the patients themselves could not complete the form due to a limitation, assistance was rendered by the patient's caregiver or clinic staff. Patients <18 years old, those completing a questionnaire at a previous visit, or those unable to comprehend the English language-based questionnaire were excluded. No patient refused participation. The survey included questions regarding patient demographics, distance traveled, transportation difficulties and the time required for the patients and their caregiver to prepare and commute to their appointments.

Following study completion, all survey information was databased in Excel. The patients commute time was calculated by combining the patients' reported travel time to and from the clinic. A minority of patients (*n* = 12) included time for their trip to the clinic but not back home. For these patients, the reported time to clinic was simply doubled. In rare cases where distance and time estimates were not answered (*n* = 14), the estimated distance from the patient's home to clinic was computed using Google maps with departure set to noon on the weekday of the clinic visit (https://www.google.com/maps). In addition to patient commute and preparation time to clinic, the study also included the time a patient family member or caregiver spent coming to clinic, when applicable (max of one person since multiple caregivers were not discernable by our survey).

## Results

There were 208 independent completed questionnaires. The median age of the population was 49.0 years with a median of 4.0 years since the onset of neurologic disability. A greater proportion of the population was male (63.5%) and the most common neurologic diagnosis in the study population was spinal cord injury (SCI) (37.0%), followed by non-traumatic brain injury (20.2%), and traumatic brain injury (13.5%), with cerebral palsy, multiple sclerosis, and spina bifida making up the remainder. The insurance status of the study population was mixed. Most patients were covered by Medicare (38.0%) or Medicaid (California Medicaid) (34.6%), with the remaining 27.3% covered by private insurance, Workers' Compensation, or in rare cases no insurance coverage. The majority of patient visits were to physiatrists (88.9%) with a minority to a neuro-urologist (13.0%) (several as joint appointments) (Table 1).

**Table 1:** Population demographics, injury characteristics, and transportation.

**Sample characteristics**	
**Characteristic**	**Median (IQR)**
Age (years)	49.0 (34.2–60.9)
Years since injury	4.0 (1.0–19.5)
**Gender**	***N*** **(%)**
Male	132 (63.5%)
Female	76 (36.5%)
**Insurance**	
Medicare	79 (38.0%)
Medicaid	72 (34.6%)
Private	38 (18.2%)
Workers' Comp	18 (8.6%)
No insurance	1 (0.5%)
Visit type^*^	
Physical Medicine & Rehabilitation	185 (88.9%)
Urology	27 (13.0%)
**Neurologic disorder**	
SCI/transverse myelitis	77 (37.0%)
Non-traumatic brain injury (stroke, tumor, other)	42 (20.2%)
Traumatic brain injury	28 (13.5%)
CP	13 (6.2%)
MS/neuromyelitis optica	5 (2.4%)
Spina bifida	5 (2.4%)
Other	20 (9.6%)
No response	18 (8.6%)
**Caregiver/family present**	
Yes	158 (76.0%)
No	50 (24.0%)
**Transport method**	
Personal car—driven by family/friend	105 (50.5%)
Personal car—patient driven	38 (18.3%)
Wheelchair van (scheduled)	32 (15.4%)
Car service (cab, rideshare)	11 (5.3%)
Public transit/bus	7 (3.4%)
Ambulance	2 (1.0%)
Other/unknown	13 (6.2%)
**Transit payor**	
Patient/family	131 (63.0%)
Insurance	34 (16.3%)
Other/unknown	43 (20.7%)
**Wheelchair use**	
Used for visit	105 (50.5%)
Not used for visit	89 (42.8%)
Unknown	14 (67.3%)

* *Patients could see more than one provider on the same day*.

*SCI, spinal cord injury; CP, cerebral palsy; MS, multiple sclerosis*.

**Figure 1:**
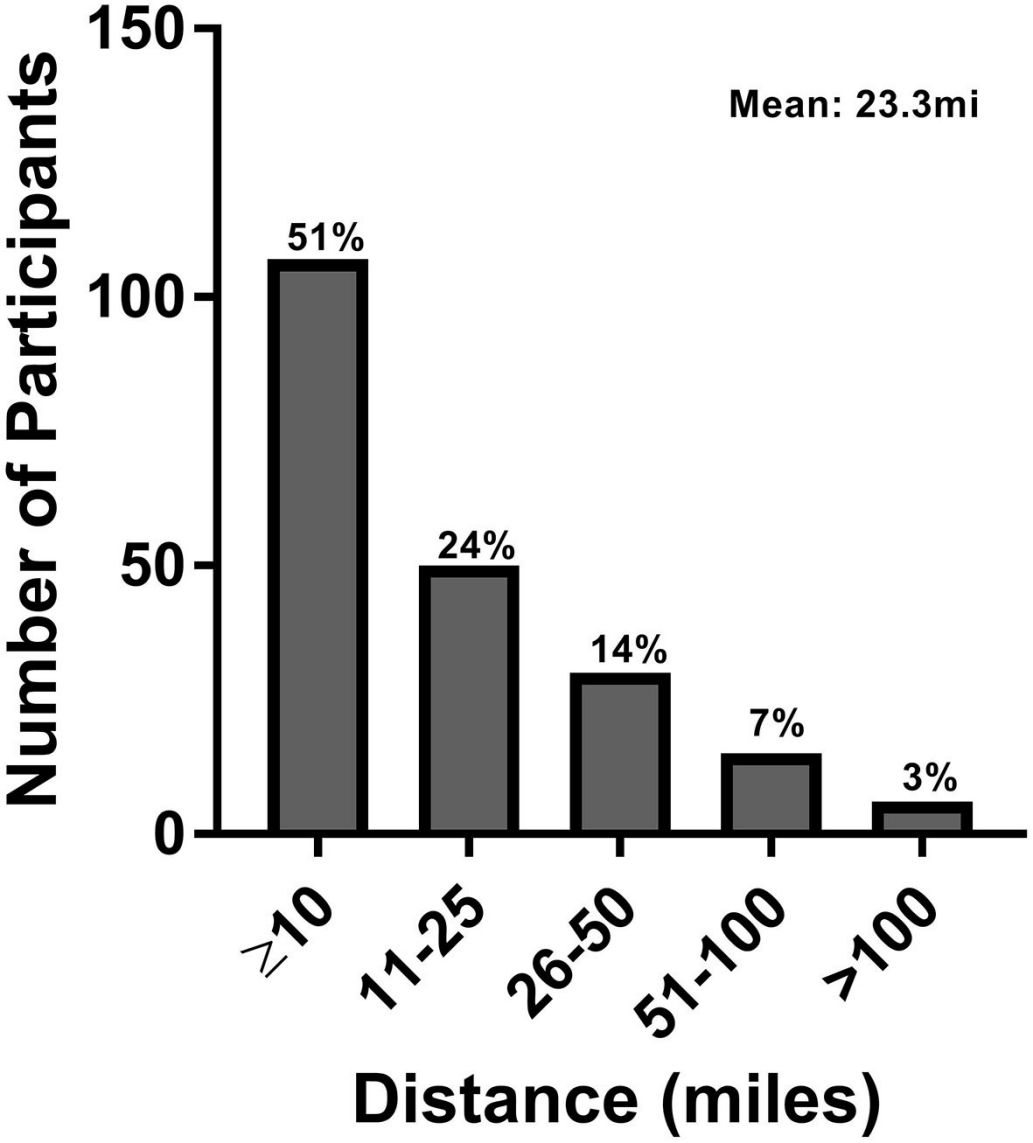
Patient distance between home and clinic.

The mean distance from a patient's home to the clinic was 23.3 miles with roughly one fourth of the population commuting >25 miles (range 0.25–315 miles) (Figure 1). Patients commuted to the clinic in a variety of ways, most commonly in a personal car driven by family or friends (50.5%), followed by patient-driven car (18.3%), and hired wheelchair van (15.4%). Two patients were brought by non-emergent ambulance. Transit costs were largely reported to be paid by patients or family (63.0%), with 16.3% reported to be paid through insurance (Table 1). Of a subgroup of patients who reported travel costs (*n* = 65), a mean cost of $39.10 (range 0–$400) was reported.

**Figure 2:**
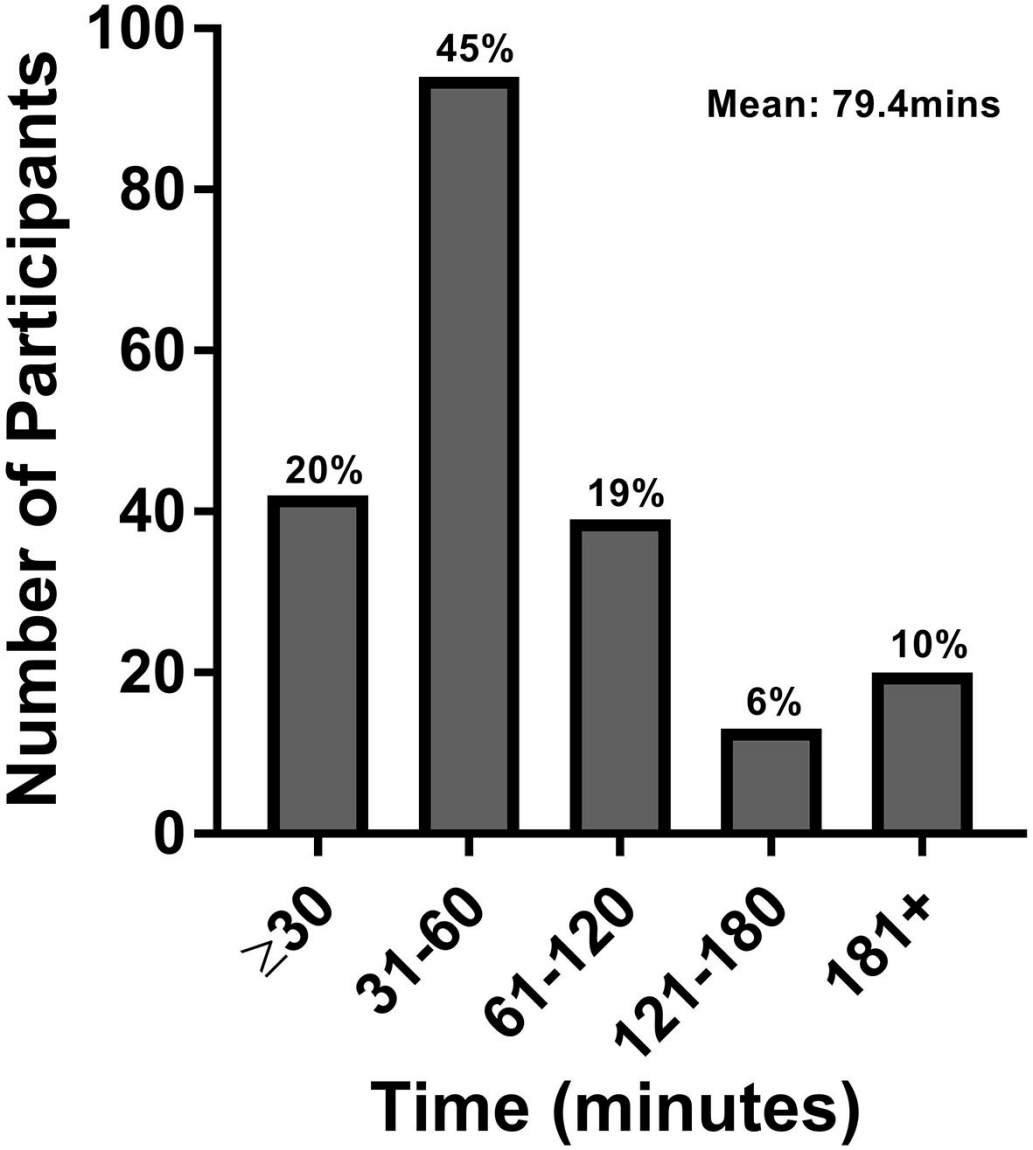
Patient commute time. This figure represents the total roundtrip commute time in minutes for patients only.

**Figure 3:**
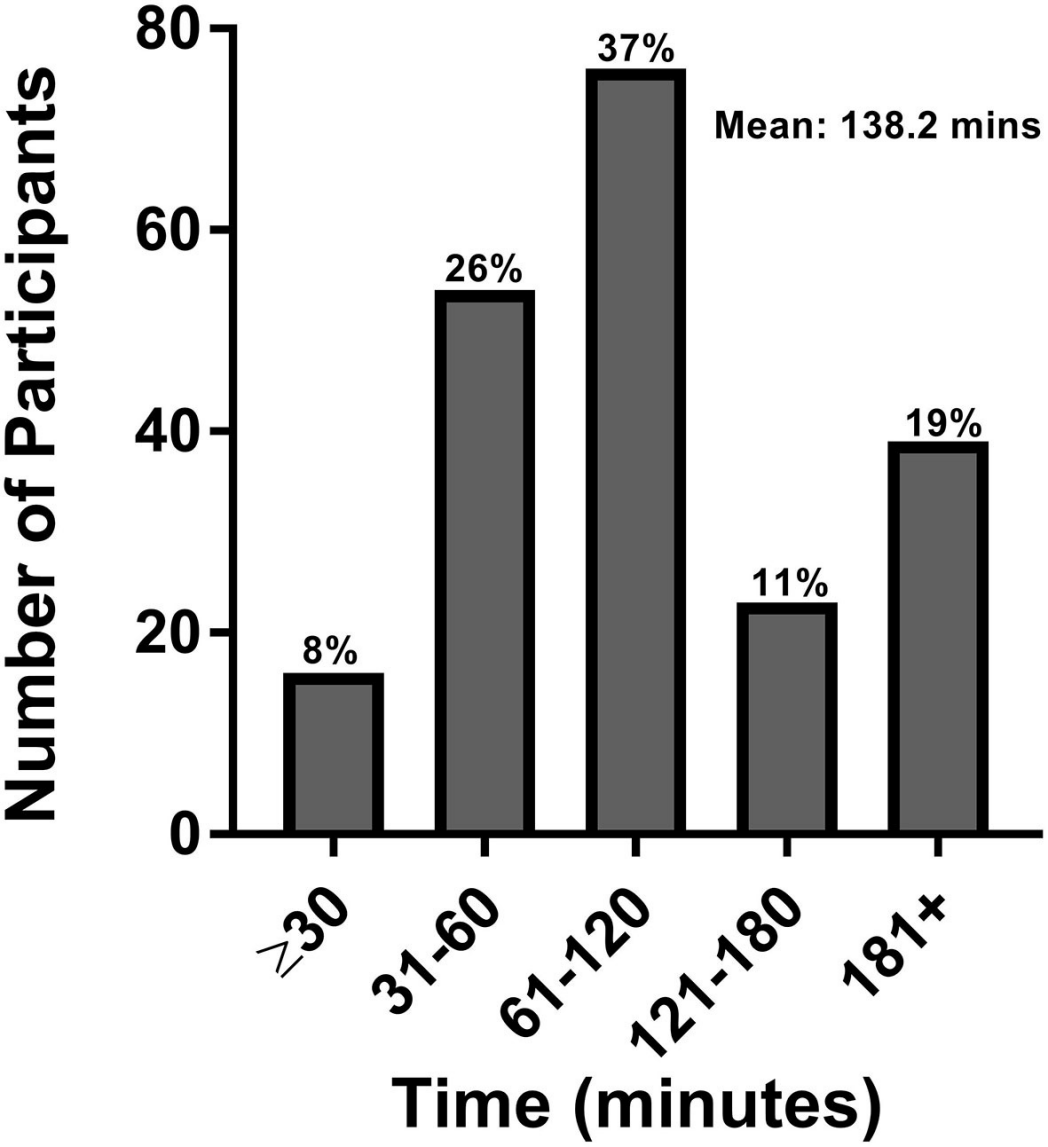
Patient and caregiver commute time. This figure represents the total roundtrip commute time in minutes for patients and caregivers.

The study population estimated that they required a mean of 79.4 min to commute to their clinic visit and back home, with 10% requiring more than 3 h of commute time (Figure 2). Patients were often accompanied to the clinic by either a family member (63.5%) or caregiver (12.5%). When accounting for the additional time spent by family or caregivers, the calculated time estimates increased to a mean total of 138.2 min commute time with 19% requiring over 3 h of commute time (Figure 3).

Roughly 40% of the study population noted difficulties coming to their clinic visit. Specific difficulties mentioned included transport itself or arranging transport (35.6%), time spent commuting to clinic (27.6%), their health condition (18.4%), parking (17.2%), changes to their daily schedule (16.1%), trouble transferring from their vehicle to a wheelchair (6.9%), and out of pocket cost (2.3%), with some patients mentioned more than one difficulty (Table 2). Of those reporting a need to make special arrangements for transportation to clinic, it was estimated that an average of 18 min was required, and the arrangements generally needed to be at least a week prior to their visit.

**Table 2:** Barriers to clinic visits.

**Difficult to come to clinic**	***N* (%)**
Yes	87 (41.8%)
No/not answered	121 (58.2%)
**Transport difficulty categories**	
Act of transport/arranging transport	31 (35.6%)
Commute time/time spent arranging transportation	24 (27.6%)
Health condition	16 (18.4%)
Parking	15 (17.2%)
Changes to schedule (work, childcare, school, caregiver)	14 (16.1%)
Transport between parking lot and clinic	6 (6.9%)
Cost	2 (2.3%)

*N count*.

## Discussion

No matter our goals in life, a key component is the time to pursue them. In persons with neurologic disorders, simple activities of daily living often are more time-consuming and inconvenient to perform. An increased need for medical care only further increases the time burden to their daily life. We find that individuals with chronic neurologic disorders require substantial amounts of commute time to and from medical visits, with the average patient requiring 79.4 min, and about 10% spending more than 3 h per visit. In addition, family and caregivers are also affected, as more than 75% of patients had a family member/caregiver present at their appointment. Taken together, the time burden of clinic visits is more pronounced, with an average total commute time of 138.2 min with ~20% requiring 3 or more hours. As we would surmise that visit times with a health care provider (whether in-person or via telemedicine) are 15 min based on clinical templates, a key difference between visit types would be the travel time.

The time burden that individuals with chronic neurologic disability and their family/caregivers face in receiving medical care can be influenced by geographic, physical, psychosocial, or transportation related barriers (5, 23–26). We found that 41.8% of patients reported difficulty in attending clinic visits. Transportation difficulties, commute time/preparation time, and changes to their daily schedule were the most commonly cited reasons (Table 2). These difficulties may disproportionately affect individuals with chronic neurological disabilities compared to abled-bodied individuals. As such, telemedicine visits may reduce these obstacles significantly (27) with prior studies demonstrating SCI patients endorsing telemedicine as easy to use, efficient and convenient with high patient perceived health satisfaction (8, 20).

To date, while many studies have shown increased patient satisfaction and comparable care outcomes with the use of telemedicine, few have focused on the convenience it offers specifically from a time perspective (8, 20, 28). Two studies of Veteran Affairs patients in Atlanta and Los Angeles with large catchment areas found an average per patient time savings of 3–4 h for those attending urology appointments (29, 30). Other studies have found a reduction in average round trip commute time of 39 min per patient for those attending vascular surgery clinics and 80 min for outpatient orthopedic surgery follow-ups (31, 32). Our findings of ~79.4 min spent commuting to and from a clinic visit in a population with chronic neurologic disorders are comparable; however, when factoring in the need for family/caregiver assistance, these estimates increase substantially. The family/caregiver component should also be viewed as an additional stress on a patient's support system where caregivers are often required to miss work to attend appointments, and caregiver burnout is prevalent (33). Easing not only the time and financial constraints but also the psychological burden on patients and their caregivers should be considered.

While some insurers now cover telemedicine encounters between a physicians' office to a patients' home/workplace, coverage is far from universal. For instance, Medicaid in certain states has restrictive requirements pertaining to patient location and distance from their provider while Medicare has yet to cover telemedicine, except in limited circumstances. As 38% of our study population falls into Medicare coverage (mostly due to chronic neurologic disability) a substantial proportion of our patients are ineligible to receive remote care at home. Prior to the recent COVID-19 outbreak, which has temporarily lifted Medicare telemedicine restrictions, it has been speculated that Medicare was reticent to widely adopt telemedicine secondary to fears of overuse and increasing costs to the system. However, there is evidence that telemedicine may decrease costs in those with chronic neurologic disorders as illustrated by a study of amyotrophic lateral sclerosis patients who utilized less home health care and had a lower risk of disease progression when using telemedicine compared to those utilizing regular clinic care at a tertiary care center (19). In addition, when one further considers the need for specialized transportation required by some of our population (16% of our sample has their transportation reimbursed by insurance), additional savings could be realized as Medicare reimbursements to medical transport companies are ~$500 for a 30-mile roundtrip in a basic life support ambulance in California's Bay Area. Further, telemedicine visits are reimbursed the same as in-office visits despite the capital outlay of electronic platforms a physician's office would need to cover. When one further considers that no-show rates of up to 17% have been documented in SCI patient visits to specialty centers, telemedicine may also improve physician efficiency and patient access, as fewer rescheduling of appointments would be required (34).

We have noted significant improvements in our ability to complete patient visits using telephone visits during the COVID-19 outbreak with a near 100% visit rate to date, and numerous patients wondering if telemedicine visits can be continued long-term. While it can be argued that telemedicine is unable to offer full physical exams, in our experience, following a patient's initial patient evaluation, most follow-up visits do not involve further physical examination. This was evidenced during the study period in the subgroup of urology patients undergoing follow-up visitation in whom 18/20 (90%) did not require physical examination; one patient needing a post-void residual check and the other a measurement of upper extremity motor function. This may argue that one can eliminate travel times and in-person visits for many patients with chronic neurologic disability, with the prospect of more worthwhile patient and caregiver experiences over their long-term care horizon.

Telemedicine is a logical extension of societal trends of digital communication with more than half of medical schools already incorporate telemedicine training into their clerkships (35). As of 2016, it is estimated that 89% of United States households have a computer or smartphone and 81% have a broadband internet subscription that will provide the basic technology to perform a telemedicine visit (36). This data may also suggest that there is a reduced burden for individuals to adopt this technology.

There are different ways to calculate the cost savings of telemedicine usage. One method is to assess the cost of time gained, from not commuting to clinic. For instance, if every patient paid for (or was given) the preferred mode of telecommunication at our institution (an Apple iPad, current cost is $329). Using average commute times, the cost to families to avoid travel and preparation time would be $2.38/min for a single visit ($329/138.2 min). There would be additional cost savings for each additional visit, such that that by the fourth visit, the cost would decrease to $0.60/min [($329/138.2 min)/4 visits]. For patients, family members, and payors, investing in a telemedicine device may pay dividends in terms of cost and alleviating burden. In addition, as future studies are undertaken, data on telemedicine cost savings and quality of life improvements for patients with chronic neurologic disability, should strengthen the argument for increased telemedicine utilization in this population.

### Study Limitations

Our study is limited by commute time data being derived from patient estimates rather than objectively measured times. More accurate commute times would start from the moment the patient began to physically prepare for a clinic visit, and would include parking time, travel time between parking structure and clinic, clinic registration and visit checkout. But, unlike prior telemedicine studies, we consider not only the time burden of the patient themselves, but other family members/caregivers, who often must accompany a person with disability. Including travel data to multiple clinics and including non-English speakers would provide more generalizable results and including patients without neurological disabilities attending clinic visits may elucidate shared challenges. In addition, the majority of this sample (75%) included individuals living within 25 miles, which may underrepresent the time burden for individuals with neurological disorders that live in rural communities or attend centers with larger catchment areas. Yet, this study was conducted at a regional specialty center of excellence and was composed of a large number of participants with a wide range of neurological conditions, which may mediate issues related to the study's generalizability.

## Conclusion

Telemedicine has the potential to substantially improve time savings for those requiring care for their chronic neurologic disorders. Increased emphasis on telemedicine coverage for those with chronic neurologic disability should be considered by all payors, especially considering the time burden that is placed not only on patients, but also their family members and caregivers who often assist them in attending specialty care visits.

## Data Availability Statement

The datasets presented in this article are not readily available because due to the nature of this study, the participants did not agree for their data to be shared. Requests to access the datasets should be directed to the corresponding author.

## Ethics Statement

Ethical review and approval was not required for the study on human participants in accordance with the local legislation and institutional requirements. Written informed consent for participation was not required for this study in accordance with the national legislation and the institutional requirements. However, written informed consent was implied via completion of the survey.

## Author Contributions

DS, BD, KS, and CE shared in the manuscript's concept, design, data acquisition, data analysis, and writing. All authors contributed to the article and approved the submitted version.

## Conflict of Interest

The authors declare that the research was conducted in the absence of any commercial or financial relationships that could be construed as a potential conflict of interest.

## Supplementary Material

Supplementary Material 1 Patient questionnaire.
