# Supplementary material for: The Time Burden of Specialty Clinic Visits in Persons With Neurologic Disease: A Case for Universal Telemedicine Coverage
Source: Front Neurol. 2021 May 4;12:559024. doi: 10.3389/fneur.2021.559024 (PMC8130896; doi:10.3389/fneur.2021.559024)
Supplement: Supplementary Material 1 — Patient questionnaire. [file Table_1.DOCX]

**Time Study**

1. Age (years) ______________________
2. Gender: Male / Female
3. Years with neurologic disorder ________________
4. Estimated distance from your home to the clinic (miles) ______________________________
5. Primary reason for your clinic visit today?
   1. To see a rehabilitation doctor
   2. To see a urologist
   3. Other ___________________________________
6. What primary issue did you come to the clinic for today?
7. Bladder problem
8. Bowel problem
9. Equipment problem
10. Medication questions
11. Other __________________________________
12. Do you have one of the following?
    1. Paraplegic (leg weakness)
    2. Quadriplegia (leg and arm weakness on both sides)
    3. Hemiplegia (leg and arm weakness only on one side)
13. What best describes your neurologic disorder?
14. Spinal cord injury
15. Transverse Myelitis
16. Spina bifida
17. Multiple Sclerosis / Neuromyelitis Optica
18. Nontraumatic brain injury (stroke, tumor, other)
19. Traumatic brain injury
20. Other – ____________________________________
21. Means of transport you used to come to the clinic today?
22. Personal car – drive yourself
23. Personal car – driven by family/friend
24. Public transit / bus
    1. Did you need to take more than 1 bus / public transit: Yes / No
    2. If yes, how many _________________________________
25. Wheelchair van (scheduled)
26. Ambulance
27. Cab service (Uber/Lyft/Cab)
28. Walk / wheelchair only
29. Other _______________________________
30. Who pays for your transportation?

Insurance

Patient

Other _________________________________

1. If you paid for transportation – what do you estimate it costs to come to the Rehabilitation clinic today (transport here and back to home)?
2. Do you use a wheelchair primarily for your mobility?

Yes / No

1. Are you in a wheelchair to come to clinic?

Yes/No

1. How long does it take you to get from your residence to the clinic where you are being seen today (minutes)? ____________________________________________________________
2. How long will it take you to get home from the clinic where you are being seen to your residence after the visit today ? (minutes) ________________________________________
3. Are you accompanied by
4. Family member
5. Friend
6. Attendant / caregiver
7. Healthcare provider (social worker, case manager, nurse, etc)
8. Other _____________________________________________
9. Are there extra preparations to get ready for a clinic visit that you do not do on a typical day?

_____________________________________________________________

1. If yes, how long does this preparation take (minutes)? ________________________________
2. How much time did you need to make ride arrangements (if applicable)? __________________
3. What makes it difficult for you to come to this clinic?
4. Not difficult
5. Other – ___________________________________________________________
